# Supplementary material for: Real-time monitoring of replication errors’ fate reveals the origin and dynamics of spontaneous mutations
Source: Nat Commun. 2024 Mar 27;15:2702. doi: 10.1038/s41467-024-46950-0 (PMC10973407; doi:10.1038/s41467-024-46950-0)
Supplement: Supplementary file 1 — Supplementary Information [file 41467_2024_46950_MOESM1_ESM.pdf]

Title: Real-time monitoring of replication errors' fate reveals the origin and dynamics of spontaneous mutations

Authors: Chiara Enrico Bena[1], Jean Ollion[2,3], Marianne De Paepe[1], Magali Ventroux[1], Lydia Robert[1,#,*], Marina Elez[1,#,*]
Affiliation: [1]Université Paris-Saclay, INRAE, AgroParisTech, Micalis Institute, 78350, Jouy-en-Josas, France.
[2]Sorbonne Université, CNRS, Institut de Biologie Paris-Seine (IBPS), Laboratoire Jean Perrin (LJP), 75005 Paris, France
[3]SABILab, Die, France

[#] equal contribution
* correspondence to: Marina.elez@inrae.fr, Lydia.robert@inrae.fr

Supplementary Information

MAIN INDEX:

Supplementary Note 1
Supplementary Note 1
Supplementary Figures
Supplementary Tables

Supplementary Note 1: Data analysis

Estimating YFP-MutL foci rate

The rate of YFP-MutL foci appearance was calculated by counting the number of newly appeared foci per segmented cell (i.e., one cell at a given time point) and dividing it by the time interval between two acquisitions (Δt). In the long-term experiments, the rate of foci is stable over time (Supplementary Figure 10), therefore the average rate was calculated by considering cells from all frames (Supplementary Table 9). In contrast, in the short-term experiments, only cells present in frames 2-6 were considered (Supplementary Table 2), as the rate of foci decreases over time due to bleaching caused by frequent illuminations (Supplementary Figure 2d).

Estimating the lifetimes of YFP-MutL foci from short-term experiments

To determine the lifetime of a focus, we multiplied the temporal resolution of the experiment (Δt) by the total number of frames in which the focus was detected. In order to prevent biases, we implemented two different exclusion criteria for foci with truncated lifetimes (i.e., foci that are present on the first or last frame): one for WT, MF1R strains and another one for *mutH*. For WT and MF1R strains, we discarded all foci present in the first frame and considered only foci appearing in the first 105 seconds of the experiment. In this way, since the experiments lasted 225 s, all truncated foci have lifetimes > 120s. They are pooled together in the last bin of the histograms in Figure 1e and Supplementary Figure 1. In *mutH* strain, YFP-MutL foci lifetimes generally exceeded the duration of the experiments, therefore we selected foci that were visible at the middle of the experiment, i.e., at 2 minutes. Truncated foci therefore have a lifetime larger than 2 minutes and are pooled in the last bin. The total number of data used in Figure 1e and Supplementary Figure 1 are shown in Supplementary Table 4.

Comparison of lifetimes of YFP-MutL foci determined from short-term experiments with different time resolutions

To compare the distributions of foci lifetimes obtained at different temporal resolutions (Δt) of 7.5 seconds or 15 seconds, we converted the measured lifetimes with Δt_{exp}=7.5 seconds into the lifetimes that would be obtained if the measurements were performed at Δt = 15 seconds. More precisely, for each detected focus, only the even frames were selected for the analysis. The converted lifetime was then calculated by multiplying the number of selected frames by 15 seconds (Figure 1e and Supplementary Figure 1). In Supplementary Figure 2c, where a third experiment performed with Δt_{exp}= 30 seconds is present, we applied the same procedure by converting lifetimes acquired with

temporal resolution of Δt_{exp} = 7.5 seconds and 15 seconds into lifetimes that would be obtained if the measurements were performed at 30 seconds.

Bleaching analysis

First the bacteria were segmented using BACMMAN, from the mCherry images as outlined in Methods subsection on image analysis. Next, the mean fluorescence intensity of the background (I_B) was computed. The I_B value was then subtracted from the mean YFP intensity of each segmented cell, resulting in the net mean cell intensity. Within the same field of view, the same I_B value was subtracted from all cells. Supplementary Figures 2a and 2b display the average net mean intensity per cell, which has been normalized to the value at time 0 (first frame of acquisition). The error bars have been obtained by propagating the error of the two averages (SEM) over their ratio. Specifically, being a and b the average net mean intensity at any frame and at frame 0 and err_a and err_b their SEMs, the normalized value (c) and its error (err_c) are given by:

$$c = \frac{a}{b} \; ; \; err_c = c\sqrt{\left(\frac{err_a}{a}\right)^2 + \left(\frac{err_b}{b}\right)^2}.$$

Estimating the loss of YFP-MutL foci appearances due to 2-minutes discrete observations

By conducting short-term experiments with a time interval of 7.5 seconds and 15 seconds between two consecutive acquisitions, we determined that the lifetime of short-lived foci in WT cells is approximately ~40 seconds. Notably, this duration is shorter than the time interval of 2 minutes between consecutive e acquisitions in the long-term experiments.

As discussed in the main text, this suggests that a significant portion of foci with lifetimes less than 2 minutes remains undetected in this type of experiment, leading to a bias in the estimation of the overall rate of YFP-MutL foci.

To correct for this bias, we used simulations to estimate the percentage of foci that are lost in these experiments.

We simulated a long-term experiment in the following way.

1. The simulated process is a Poisson process with a rate (r = 0.065 min^{-1}) obtained by averaging the rate of YFP-MutL foci lasting less than 2 minutes and detected from three short-term WT experiments. The duration of the process T_{obs}, is drawn from the distribution of lineage durations observed in three long-term WT experiments combined (Supplementary Figure 4a).

2. Each simulated focus has a duration drawn from the empirical distribution of foci lifetimes. This distribution is obtained by pooling the lifetimes of foci lasting less than 2 minutes from the three short-term WT experiments (Supplementary Figure 4b).

3. The observations are discrete and taken at regular intervals of time dt.

The fraction of observed foci can then be calculated. It is represented as a percentage in Supplementary Figure 4c.

We varied dt and we ran 500 simulations for each dt chosen. The chosen dt values, along with the average and twice the standard error of the mean of the simulations for each condition are shown in Supplementary Figure 4c.

Our results show that for dt = 2 minutes (as in our long-term experiments), the fraction of observed events is (34.07 ± 0.02) %.

This allows us to correct for the loss of short-lived foci in our WT long-term experiments.

To correct the rates of YFP-MutL foci in *mutH* pMutH cells growing in LB with and without 0.05% of arabinose (*mutH* pMutH ara 0.05% and *mutH* pMutH, respectively), we performed the same simulations as described for WT, with a slightly different distribution for the lifetimes of YFP-MutL foci. Indeed, Figure 3a shows that the lifetime of short-lived foci (< 16 minutes) gets slightly broader when the amount of MutH decreases. Therefore, we computed the ratio of the average lifetime of these short-lived foci for *mutH* pMutH and *mutH* pMutH ara 0.05% with respect to WT (Supplementary Table 6). Then, i) we multiplied the lifetimes of short-lived foci shown in Supplementary Figure 4b by these factors, ii) we discarded those lasting more than 2 minutes and finally obtained the new distributions of foci lifetimes used to simulate the loss of foci in long-term experiments for *mutH* pMutH and *mutH* pMutH ara 0.05%. According to our simulations, the percentages of detected foci in long-term experiments is (50.19 ± 0.04) % and (39.86 ± 0.03) % of the total errors produced, for *mutH* pMutH and *mutH* pMutH ara 0.05% respectively (Supplementary Table 6).

In Supplementary Figure 4d, the corrected rates (in red) for WT, *mutH* pMutH ara 0.05%, and *mutH* pMutH cells are shown to fall within the range of variability observed in:

i) the rate of all foci in *mutH* cells detected during long-term experiments, where every occurrence of foci is captured.

ii) short-term experiments, represented by the shaded grey area, which was determined as the average +/- 2 standard deviations of the YFP-MutL foci rates in the WT, *mutH*, and MF1R grown in the absence of arabinose.

Method to determine the threshold between short and long-lived YFP-MutL foci.

To determine the threshold between short and long-lived YFP-MutL foci, we used the bimodal distribution of YFP-MutL foci lifetimes observed in *mutH* pMutH grown in LB (cyan distribution in Figure 3a), that we model as a mixture of an exponential distribution (for short-lived foci) and gaussian distribution (for long-lived foci).

The procedure to determine the threshold value involves the following steps.

1. An initial guess (thr_0) for the threshold (thr) allows to divide the foci into two groups of n short-lived foci and m long-lived foci. Foci lasting exactly the value of the threshold were considered short-lived.

2. The parameters of the exponential and gaussian distributions and their normalizations are computed as follows:

- The parameter of the exponential distribution: is given by $l_{exp} = 1/\bar{x}$, where $\bar{x}$ is the mean of the lifetimes of short-lived foci, and $\frac{n}{n+m}$ is the normalization coefficient (the fraction of the foci that are short-lived).
- The parameters of the gaussian distribution are the mean and standard deviation of the lifetimes of long-lived foci and $\frac{m}{n+m}$ is the normalization coefficient.

3. The intersection point between the normalized exponential and normal distributions obtained in step 2 is calculated analytically. If multiple roots are found, the smallest root is chosen and rounded to the nearest integer. This becomes the new threshold (thr_{new}).

4. If thr_{new} is different than thr_0, thr is updated to thr_{new}, and the procedure (steps 1 to 4) is repeated until it converges (thr $=thr_{new}$).

We applied this procedure to the combined data from three *mutH* pMutH experiments, and found a threshold of 7 frames, i.e., 14 minutes (Supplementary Figure 5). The result is robust to the assumption of an exponential distribution for short-lived foci.

Estimating the mutation rate from YFP-MutL foci

The mutation rates shown in Figure 3b and Supplementary Figure 6, were obtained from the rate of long-lived YFP-MutL foci detected in the mother cells of the long-term experiments. First, we converted the rate of long-lived YFP-MutL foci appearance per minute (see the first section of this Supplementary Note 1) to a rate per generation by multiplying the per minute rate for each experiment by the average doubling time of the cells specific to that experiment. Second, since long-lived YFP-MutL foci do not represent mutations but replication errors that will be converted into mutations, it

is necessary to consider the impact of DNA segregation and multifork replication to estimate the mutation rate [1]. Accounting for these factors, the proportion *p* of mutant cells in the progeny of a cell exhibiting a long-lived YFP-MutL focus can be calculated. For *E. coli* cells with an average doubling time of 26 minutes, this proportion was determined to be 0.12 [1]. The mutation rate is calculated by multiplying the per generation rate of long-lived YFP-MutL foci by *p*.

Estimation of the kinetics of error repair from short-term time-lapse experiments

As explained in the second section of this Supplementary Note 1, in order to avoid a bias caused by truncated focus tracks (i.e., foci that appear towards the end of the experiment and cannot be followed until they disappear), all the lifetimes larger than 2 minutes are pooled together. They represent ~2% of the data, and one half of them represent unsuccessful repair (long-lived foci). However, they do not significantly bias the estimation of repair time since excluding the lifetimes larger than 2 minutes or including them has a negligible impact of ~3% on the estimated average repair time.

A variation of +/- 2 minutes in the threshold value on foci lifetimes does not alter the conclusions on the mutation rates and the temporal dynamics of long-lived foci.

We applied our procedure for finding the threshold on 5 different experiments of *mutH* pMutH with or without arabinose 0.05%, and found a variation of only 1 frame (i.e., 2 minutes). Variations within this range does not impact any of the conclusions presented in this study. Below, we show this specifically for the results presented in Figure 3b and Figure 6d.

In Supplementary Figures 6 and 13 we define long-lived foci as characterized by lifetimes lasting more than 12 minutes, 14 minutes and 16 minutes and we show that in all cases:

i) the comparisons of the mutation rate obtained from the rate of the long-lived YFP-MutL foci is compatible with that obtained with MA+WGS (Supplementary Figure 6).

ii) the disagreement between the empirical CDF of long-lived foci and its analytical prediction holds (Supplementary Figure 13).

YFP-MutL foci in cells with varying levels of Dam and MutH.

The *dam* cells and cells overproducing Dam exhibit broader cell length distributions compared to the WT strain and all the other strains we studied. We have previously shown that larger cells have higher numbers of YFP-MutL foci [1]. In order to compare the YFP-MutL foci among WT cells, Dam overproducing cells, MutH overproducing cells, and *dam* cells, we performed the analysis on cells with similar growth rates and cell lengths. To achieve this, we eliminated from our datasets all the

cells, together with their mother and first daughter, that did not satisfy the following criteria during their entire cell cycle: (i) cell length smaller than ($\mu + 3\sigma$) = 10.3 µm, where μ and σ represent the mean and standard deviation of the cell length distribution observed in three pooled WT experiments and (ii) growth rate within the range [μ - 3σ, μ + 3σ], where μ and σ represent the mean and standard deviation of the cell growth rate distribution in three pooled WT experiments. The distributions of selected cell lengths and growth rates are presented in Supplementary Figure 7, along with the distributions of YFP-MutL focus lifetimes for all samples. The application of a more stringent threshold, which involved filtering both the growth rate and cell length based on the average of the distributions +/- 2σ, did not alter the conclusions (Supplementary Figure 8).

YFP-MutL foci in cells displaying either successful or failed repair.

For this analysis (Figure 5a), cells from three WT long-term experiments were categorized into two groups:

1) Cells with a long-lived focus: for each long-lived YFP-MutL focus detected, an interval of 3 frames before and after its appearance has been considered and the rate of all foci within such 7-frames (14 minutes) interval has been computed.

2) Cells with at least one short-lived focus: is the analogue of 1) but with the intervals +/- 3 frames centred on the appearance of a short-lived focus.

When computing the rates for each experiment we subtracted the contribution of the focus that determines the window. The values of all the intervals of each category were pooled to calculate the final rate. The total number of long-lived foci were: 202, 163 and 145.

The total number of short-lived foci were: 3592, 3896, 3325.

Repair efficiency over replicative age.

As in Wang, Robert et al. [2], we defined the replicative age as the number of consecutive generations or cell divisions of the mother cell. For each WT experiment and generation, we calculated the average rate of all YFP-MutL foci (Supplementary Figure 10a), the rate of long-lived YFP-MutL foci (Supplementary Figure 10b), and the ratio of the rate of long-lived YFP-MutL foci to the rate of all YFP-MutL foci (Figure 5b), which represents repair efficiency. We considered for this analysis all the lineages of mother cells and limited to 100 generations those lasting more than that. The total number of analysed cells and foci per experiment are shown in Supplementary Table 1.

Bias correction in the distribution of inter-arrival times of long-lived foci

We aim to characterize the dynamics of the occurrence of long-lived YFP-MutL foci. This process has an average rate of one event every 100 generations, and our observation windows last approximately 100 generations. Also, not all lineages are observed for the entire 100 generations. Consequently, for more than half of the foci, we cannot observe the following focus and therefore we are unable to calculate the inter-arrival time

To illustrate this situation, Supplementary Figure 11a provides a schematic representation. The black rectangular boxes represent $N = 4$ lineages ($M_{i=1,..4}$) observed within time windows [0, $W_{i=1,..4}$]. During these windows, only a few events (orange dots) occur at specific times t_{ij} (i = 1,..4; j = 1,..n). In M_1, 2 foci are observed but only one inter-arrival time can be calculated ($d_{1,1}$, red segment), while the other $d_{1,2}$ cannot be calculated since we do not observe any third focus. Not considering truncated intervals like $d_{1,2}$ would lead to a bias in the estimated distribution, since small inter-arrival times are more likely to be observed than long ones. In order to correct for this bias, we developed a method based on the cumulative distribution function (CDF). The CDF represents the probability that a variable, in our case, the inter-arrival times d, is less than or equal to a given value x. For estimating the CDF in an unbiased way, the principle is for each value x to compute CDF(x) on a restricted dataset corresponding to the time window [0, W - x], since for any focus appearing in this restricted time window we know if the inter-arrival time with the following focus is smaller or larger than x (in which case the following focus might not be observed). We detail the method below. We take into account i) all foci occurrences, including those not followed by another focus within time W, and ii) the duration of each of the observation time-windows W_i.

Since our experimental time is discrete, with images captured every 2 minutes, we constructed CDF(x) by incrementing x in steps of 2 minutes. Supplementary Figure 11a shows a schematic representation of the bias correction procedure. Each M_i is characterised by i) its duration W_i, ii) the times $t_{i,n}$ at which each of the n_i foci occur and iii) their corresponding inter arrival times $d_{i,n}$. For each value of x, and each lineage where $W_i > x$, we define the restricted time window as [0, $W_i - x$]. We then compute two quantities for each lineage M_i:

- $\tau_{i,x}$: the number of inter-arrival times such that $0 \leq t_{i,n} \leq (W_i - x)$,
- $\delta_{i,x}$: the number of these inter-arrival times smaller than x ($d_{i,n} \leq x$).

Next, we sum these quantities on all the lineages: $T_x = \sum_{i=1}^{N} \tau_{i,x}$ and $D_x = \sum_{i=1}^{N} \delta_{i,x}$.

Finally, the corrected CDF of the inter arrival times is given by $P(d \leq x) = \frac{D_x}{T_x}$.

In this way, the total number of foci considered is different per each value of x.

To validate this method, we performed simulations. In order to mimic our experimental conditions, we simulated 300 independent Poisson processes of the same rate on time windows of variable lengths with the following characteristics i) the rate of the Poisson process matches the average rate of long-lived YFP-MutL foci in our three wild-type experiments ($\Lambda = 0.00047$ min^{-1}), ii) the duration of the observation windows (W_is) is drawn from the distribution of lineage durations observed in our experiments (Supplementary Figure 4a) and iii) the effect of discrete observations with time intervals of 2 minutes is taken into account by approximating the times of occurrences t_i as $\lceil t_i \rceil + \lceil t_i \rceil \ mod \ 2$. To assess the effectiveness of the bias correction, we compared the biased CDF computed in a classical way, discarding truncated inter-arrival times (Supplementary Figure 11b in blue), and the corrected CDF (Supplementary Figure 11b in grey) of the simulations with the analytical expectation of the CDF for a Poisson process with rate Λ and that accounts for discrete observations performed every 2 minutes (Supplementary Figure 11b in black) [1]. The results, as shown in Supplementary Figure 11b, indicate that the analytical prediction does not agree with the biased CDF. However, the average of the corrected CDF closely overlaps the analytical expectation. This finding shows that our procedure corrects the bias efficiently. By applying the proposed correction method, we were able to compare CDF of the experimentally obtained inter-arrival times, after correction, with the analytical expectation for a Poisson process (Figure 6d and Supplementary Figure 11c).

Confidence intervals for the Cumulative Distribution Function (CDF) of YFP-MutL long-lived foci inter-arrival times in WT cells.

For the CDF of the inter-arrival times of YFP-MutL long-lived foci corresponding to the 3 experiments in WT cells (Figure 6d and Supplementary Figure 11c), we built confidence intervals for each CDF by bootstrapping. To do so, for each experiment (dataset containing N inter-arrival times), we i) performed 500 resamplings, each one consisting in sampling N inter-arrival times with replacement in the original dataset, ii) for each of these 500 datasets we computed the corrected CDF as described in the previous section, and finally iii) we calculated the 95% confidence interval corresponding to the 500 CDFs (shaded areas in Supplementary Figure 12).

Supplementary Note 2: MA+WGS

Mutation accumulation (MA) lines

MA lines were initiated by diluting $5*10^8$ initial liquid cultures grown to saturation in LB medium supplemented with 1mM IPTG, ampicillin (50 µg/ml) and arabinose (0.05-0.5%) as specified in the manuscript text and/or figure legends. 2 ml aliquots of the initial cultures were collected and stored at -70°C for extraction of genomic DNA and sequencing. Procedures for genomic DNA extraction and sequencing are described in Methods subsection dedicated to MA+WGS. At least 4 MA lines were propagated per strain and condition by diluting $5*10^8$ -fold each day, for 4 days, with the exception of strain MF1RmutS under 0.5% arabinose which was propagated for 2 days, as it accumulated >100 mutations per cycle. To estimate the number of generations in each cycle, appropriate dilutions of the saturated cultures were spread on LB plates. Details on the estimation of the number of generations can be found in the following section. On the last day, one colony from each strain was resuspended in 5 ml of LB medium (supplemented with 1 mM IPTG) and grown at 37°C until reaching an optical density at 600 nm (OD600) of 0.6 to 1. From each culture, 2ml aliquot was collected and stored at -70°C for extraction of genomic DNA and sequencing.

Estimation of the number of generations

The number of generations per cycle was estimated using the dilution factor, where the final volume was 5 ml and the initial volume was 10^{-8} ml, resulting in a dilution factor of $5*10^8$. To convert the dilution factor to generations, we applied the formula Ln(dilution factor)/Ln(2), which gave us 29 generations per cycle and 116 generations overall per line (4 cycles * 29). To validate this theoretical value, we also estimated the initial and final number of cells in each culture at each cycle by plating dilutions of saturated cultures on LB plates and counting colonies after overnight growth at 37°C. By applying the formula Ln(final number of cells/initial number of cells)/Ln(2), we obtained an estimate of the number of generations. Across all strains and conditions, the mean (± standard deviation) of Ln(final cell number/initial cell number)/Ln(2) was (29 ± 1) generation.

Mutation calling

We used an open-source computational pipeline called breseq (versions 0.36.0; [3]) to predict mutations in the samples based on how Illumina reads align to each position in the *E. coli* MG1655 genome (GenBank: NC_000913). Base substitutions and short insertions and deletions (≤ 2bp) are predicted from read alignment information as described in Deatherage et al. [3]. Larger deletions and insertions are detected using information on missing coverage and the identification of new junctions, but these

changes were ignored in our study. Additionally, breseq has limitations in predicting genetic changes associated with sequence repeats in the reference genome longer than the read length, due to constraints inherent in short-read DNA sequencing data and the algorithms employed. Consequently, such changes were also disregarded. The reference genome sequence file in GenBank format, NGS read files for genomic DNA samples in FASTQ format, and lists of predicted mutations in each evolved genome (in HTML format) are freely accessible at https://doi.org/10.57745/UFHTYU.

Estimation of the mutation rate

To estimate the per generation mutation rates for each strain and condition, we performed the following calculations for each MA line:

i) the number of mutations of the different strains used relative to the *E. coli* MG1655 sequence (GenBank: NC_000913), before the experiment, denoted as m_i

ii) the number of detected mutations at the end of the experiment relative to the *E. coli* MG1655 sequence (GenBank: NC_000913), denoted as m_f

iii) the total number of generations over the experiment, denoted as g

The mutation rate was then calculated as $\mu = (m_f - m_i)/g$.

The bias created by natural selection in our MA-WGS protocol is small

Our MA-WGS protocol is based on liquid cultures, in contrast to the classical protocol which uses solid media [4]. In principle, serial dilutions in liquid cultures can lead to deleterious mutations being purged and therefore a bias in mutation rate estimation. In order to minimize this potential bias, we perform strong dilutions, leading to a very small initial number of cells per culture (~10). We reasoned that the bias should therefore be small, since spontaneous mutations that substantially affect fitness are rare [1]. In agreement with this assumption, we obtain for an MMR-deficient strain a similar mutation rate as obtained by others with the classical protocol (our result: 0.17+/-0.021 per generation, Lee et al. [4]: 0.15+/-0.071 per generation). In order to further validate our assumption, we performed numerical simulations of MA experiments, where population size varies from 10 to 5.10^9 (as in our protocol) in each growth phase. At the end of each growth phase, 10 cells are selected at random. Mutations were generated according to a Poisson process (rate: 0.17 mutations per generation), and their effects on growth rate followed a distribution compatible with our estimation in Robert et al. [1] (1% of lethal mutations and the rest following a beta distribution of parameters 0.0074 and 2.4). We found that the bias due to natural selection in these conditions impacts mutation rate estimation by less than 5%, which is smaller than the precision of our estimation (Figure 3b).

Supplementary Note 3: Strains

For all experiments, we used the wild-type *E. coli* MG1655 (CGSG 6300) strain and its derivatives listed in Supplementary Table 11. The genotypes of the strains were verified through PCR, sequencing, and/or phenotypic assays, such as expression of fluorescent proteins or testing for the capacity to generate mutations conferring resistance to rifampicin.

To construct the strain **MVEC197**, which expresses the *dam* gene from the PLtetO1 promoter integrated into the prophage P21 attachment site, we employed the Clonetegration method [5].

Here is the overview of the construction process:

Construction of the PLtetO1-dam fragment: The *dam* gene was PCR amplified from the *E. coli* MG1655 strain. The forward primer (5'-ACGTAGAATTCTCCCTATCAGTGATAGAGATTGACATCCCTATCAGTGATAGAGATACTGAGCACATCAGCAGGACGCACTGACCAAAGAGGAGAAATTAAGCATGAAGAAAAATCGCGCTTT-3') included an EcoRI restriction site (underlined), the PLtetO1 promoter sequence and the g10 Ribosome Binding Site at the N-terminal. The reverse primer (5'-CTGCCCTGCAGATTCTCACCAATAAAAAACGCCCGGCGGCAACCGAGCGTTCTGAACAAATCCAGATGGAGTTCTGAGGTCATTACTGGATCTATCAACAGGAGTCCAAGCTCAGCTAATTAAGCTTATTTTTTCGCGGGTGAAA-3') included a PstI restriction site and a lambda T0 terminator on the C-terminal. PCR fragments were sequenced using three different primers (5'-TAGAGATACTGAGCACATCA-3', 5'CTTCGCGGAACTGATAG-3' and 5'-GCATCCGTCGTCTATTG-3'), and gel purified prior to restriction digestion.

Cloning of PLtetO1-dam fragment: PLtetO1-dam fragment was digested by EcoRI and PstI and cloned into the pOSIP-KT plasmid, which contains attP21 and P21 integrase. This resulted in the plasmid pOSIP-KT-PLtetO1dam.

Transformation and integration: The pOSIP-KT-PLtetO1dam plasmid was transformed into competent TOP10F' *E. coli* cells using kanamycin selection (20 µg/ml) at 30°C. The integration of pOSIP-KT-PLtetO1dam into the attP21 site on the chromosome was confirmed by PCR as described by St.-Pierre et al., [5]. The cloned PLtetO1-dam fragment was sequenced once again to ensure that no mutations had appeared during the different cloning steps.

P1 transduction and transformation: The pOSIP-KT-PLtetO1dam fragment, including the integration module with the kanamycin resistance marker, was transduced into the 63ME120R strain using P1 transduction. The integration was carried out at 30°C to repress the integrase from the POSIP plasmid. This resulted in the strain MVEC195.

Excision of the integration module containing the antibiotic marker: The strain MVEC195 was transformed with the temperature-sensitive pE-FLP plasmid, which expresses the FLR recombinase. The transformation was selected on ampicillin at 30°C. Clones were then streaked on LB agar and incubated at 37°C overnight to eliminate the pE-FLP plasmid. The successful excision of the integration module and elimination of the pE-FLT plasmid were verified by checking the sensitivity of selected clones to kanamycin and ampicillin as described by St.-Pierre et al., [5]. After these steps, the resulting strain was designated as MVEC197.

Strain **MF1R** expresses *yfp-mutL* from the Plac promoter, *tdCherry* from the PRNA1 promoter and *dnaQ926* allele from the P_{BAD} promoter. It is derived from the MF1 strain we previously constructed [1], which expresses the *dnaQ926* allele from the P_{BAD} promoter, integrated into the P21 prophage attachment site. To generate MF1R, several steps were performed. First, we employed P1 transduction to transfer the *yfp-mutL-cam* construct, which integrates into the lacZ locus, from the ME120 strain [6] into MF1. This step led to the creation of strain MF3. Next, we performed another P1 transduction, to transfer the *mutL::kan* from MG1655mutL [1] into MF2. This step resulted in strain MF6. To remove the CamR and KanR cassettes, site-specific recombination was performed between FRT sites using the pCP20 plasmid, following the method described by Datsenko et al. [7]. This yielded strain MF7. In the final step, we inserted the *tdCherry* gene into the MF7 strain between two Tn7 insertion sites. To accomplish this, we used the pNDL-32 plasmid obtained from Johan Paulsson's lab, following the protocol described previously [1]. The pNDL-32 plasmid, which carries the *tdCherry* gene, is temperature-sensitive and confers ampicillin resistance. After transforming pNDL-32 into MF7, we selected for transformants on LB agar plates supplemented with 100 μg/mL ampicillin. To eliminate the plasmid, we streaked the transformants twice: once at 30°C and once at 42°C, on LB agar. We verified successful integration and plasmid loss by examining selected clones for two characteristics: red staining and ampicillin sensitivity. The resulting strain was named MF1R.

To generate strain **63ME134R**, which expresses *yfp-mutL* from native *mutL* promoters and *tdCherry* from the PRNA1 promoter, the following steps were performed. First, we employed P1 transduction to transfer the *mutL::yfp-mutL-cam* construct from the ME131 strain [8] into the MG1655 6300 strain. This led to the creation of strain ME6314, where the *mutL* gene was replaced with the *yfp-mutL-cam* construct. Following that, we introduced the *tdCherry* gene into strain ME6314 using the pNDL-32 plasmid and the same protocol as described above for the construction of strain MF1R. Through these steps, we generated strain 63ME134R.

Supplementary Figures

Supplementary Figure 1. Distribution of lifetimes of YFP-MutL foci in short-term experiments.

Equivalent of Figure 1e, but showing all experiments together. The distribution of lifetimes of YFP-MutL foci in *mutH*, WT and MF1R strain under different levels of induction of the P_{BAD} promoter. The experiments were performed with time intervals of 7.5 seconds and 15 seconds. In *mutH* and MF1R cells YFP-MutL was expressed from the P_{lac} promoter, while in WT cells, it was expressed either from the P_{lac} promoter (WT) or the native MutL promoter (WT Pnat). To enable comparison, the distributions are converted as if all experiments were acquired with a time interval of 15 seconds (Supplementary Note 1 section on YFP-MutL lifetimes comparisons). Lifetimes exceeding 120s are grouped into a single bin. See Supplementary Table 4 for the number of foci lifetimes in each distribution. Source data are provided as a Source Data file.

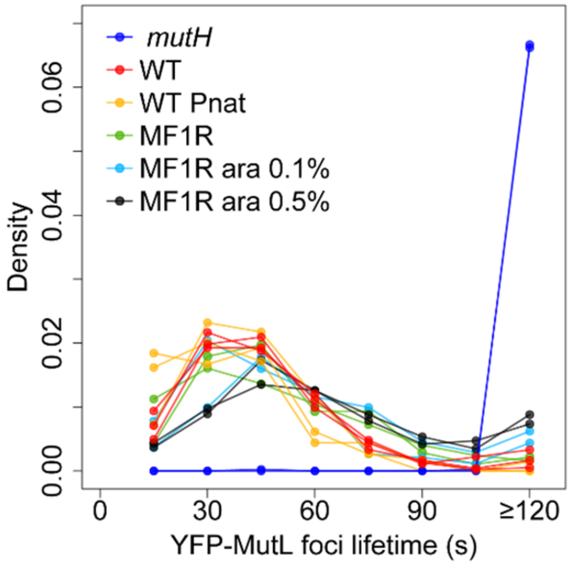

Supplementary Figure 2. Effect of bleaching on the YFP-MutL fluorescence intensity and the lifetime of foci.

a) Bleaching curves of YFP fluorescence intensity in WT cells from short-term experiments with different time resolutions: 7.5 seconds (red), 15 seconds (cyan), and 30 seconds (blue). The dots represent the average YFP net fluorescence intensity per cell normalized to the value at frame 0 calculated over all cells at a specific frame. The procedure to obtain error bars is detailed in Supplementary Note 1 section on bleaching (when smaller than dots the error bars are not visible). The data are from one representative experiment for each condition. **b)** Bleaching curve of YFP fluorescence intensity in WT cells from one representative long-term experiment with time resolution of 120 seconds. Normalized net YFP fluorescence as well as error bars were calculated as in a). **c)** The distributions of YFP-MutL foci lifetimes corresponding to the experiments shown in a). We performed the analysis as if all experiments were performed with a time resolution of 30 seconds (Supplementary Note 1 section on YFP-MutL lifetimes comparisons). **d**) Rate of YFP-MutL foci as a function of time for the WT experiment (Δt 7.5 seconds) shown in a) and c). Source data are provided as a Source Data file.

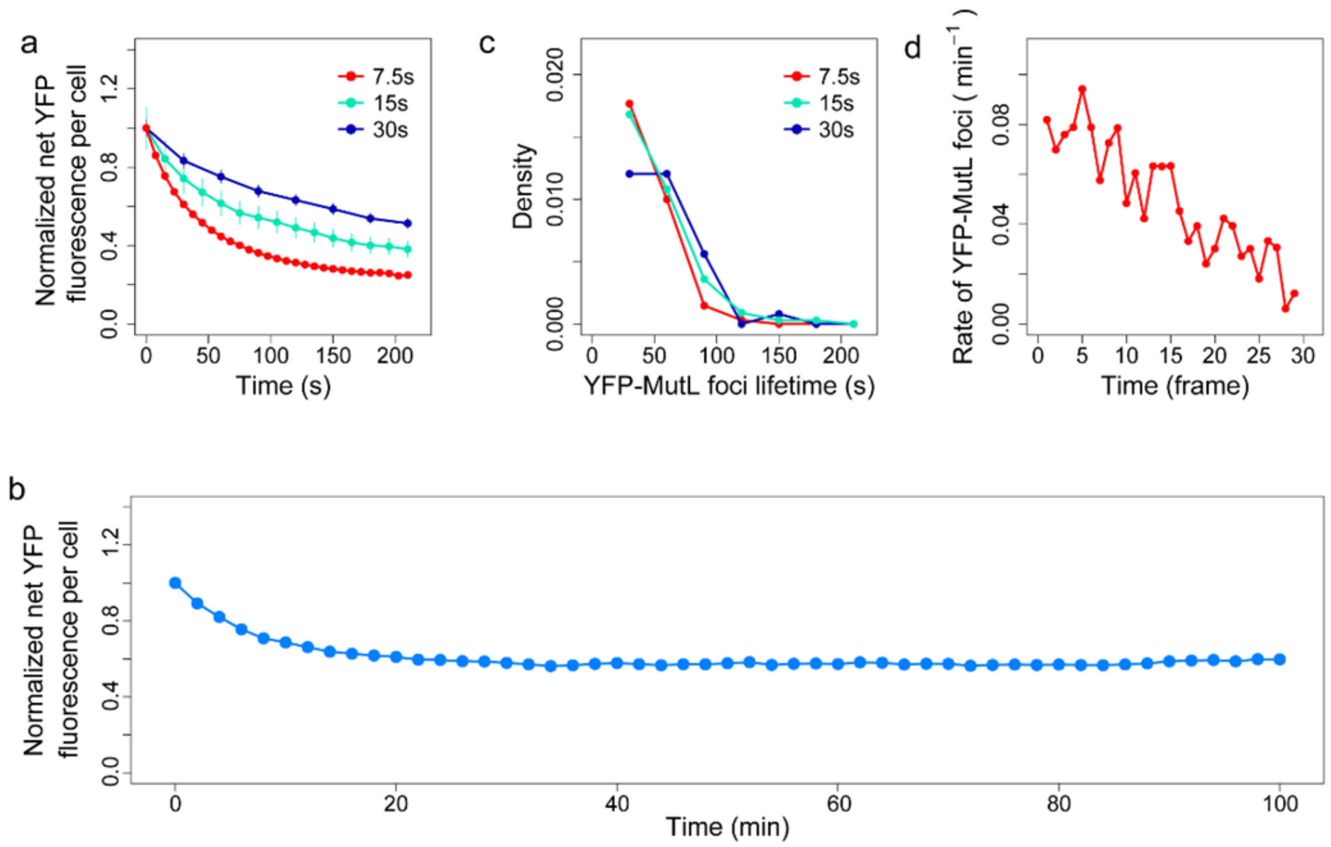

Supplementary Figure 3. Increase in the production of errors by induction of DnaQ926 in MF1 strain.

Replication error rate, determined by estimating the mutation rate through whole genome sequencing of mutation accumulation lines (MA+WGS) performed on *mutS* and MF1R*mutS.* The bars represent the average values of several experiments (dots). See Supplementary Table 5 for comparisons of the different conditions. Source data are provided as a Source Data file.

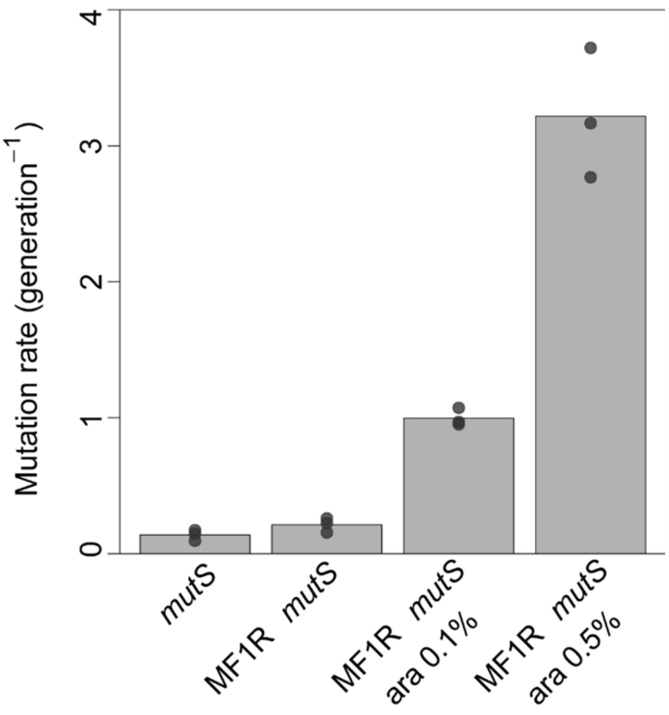

Supplementary Figure 4. Estimation of YFP-MutL foci rate from long-term experiments.

Overview of the main steps of the correction procedure to determine the effective rate of YFP-MutL foci from long-term experiments (see dedicated section in Supplementary Note 1). **a)** The distribution of lineage durations for three pooled long-term experiments in WT cells (Total number of lineages = 684). **b)** The distribution of lifetimes of YFP-MutL foci lasting less than 2 minutes for three pooled WT short-term experiments performed with a time interval of 7.5 seconds. (Total number of foci lifetimes = 150). **c)** Results of the simulations performed to determine the fraction of detected events in WT cells, for observations at different time intervals (dt). Each dot is the mean over 500 simulations. The error bars are smaller than the dots and are twice the standard error of the mean (2*SEM). Simulation parameters: i) rate $r = 0.065$ min^{-1}, ii) the duration of each simulation was drawn from the distribution presented in a) iii) the lifetimes of occurred events were drawn from the distribution shown in b). **d)** The corrected rate of all YFP-MutL foci for dt = 2 minutes in WT cells, *mutH* pMutH cells grown in LB+0.05% arabinose (*mutH* pMutH ara 0.05%) and *mutH* pMutH cells grown in LB (*mutH* pMutH) was compared to the rate of all YFP-MutL foci in *mutH* cells. Since most YFP-MutL foci lifetimes in *mutH* cells are much longer than 2-minutes, all YFP-MutL foci are considered detected in this case. The correction was performed considering that the number of detected events represents 34% (WT), 40% (*mutH* pMutH ara 0.05%) and 50% (*mutH* pMutH) of the total events (i.e., all foci lasting less than 2 minutes) (Supplementary Table 6). The experimentally obtained values are shown in black, the corrected values are shown in red. The grey shaded area represents the range of variability (mean +/- 2*standard deviations) in the rate of all YFP-MutL foci detected in short-term experiments with the WT, *mutH*, and MF1R strain grown in the absence of arabinose (Supplementary Table 2). Source data are provided as a Source Data file.

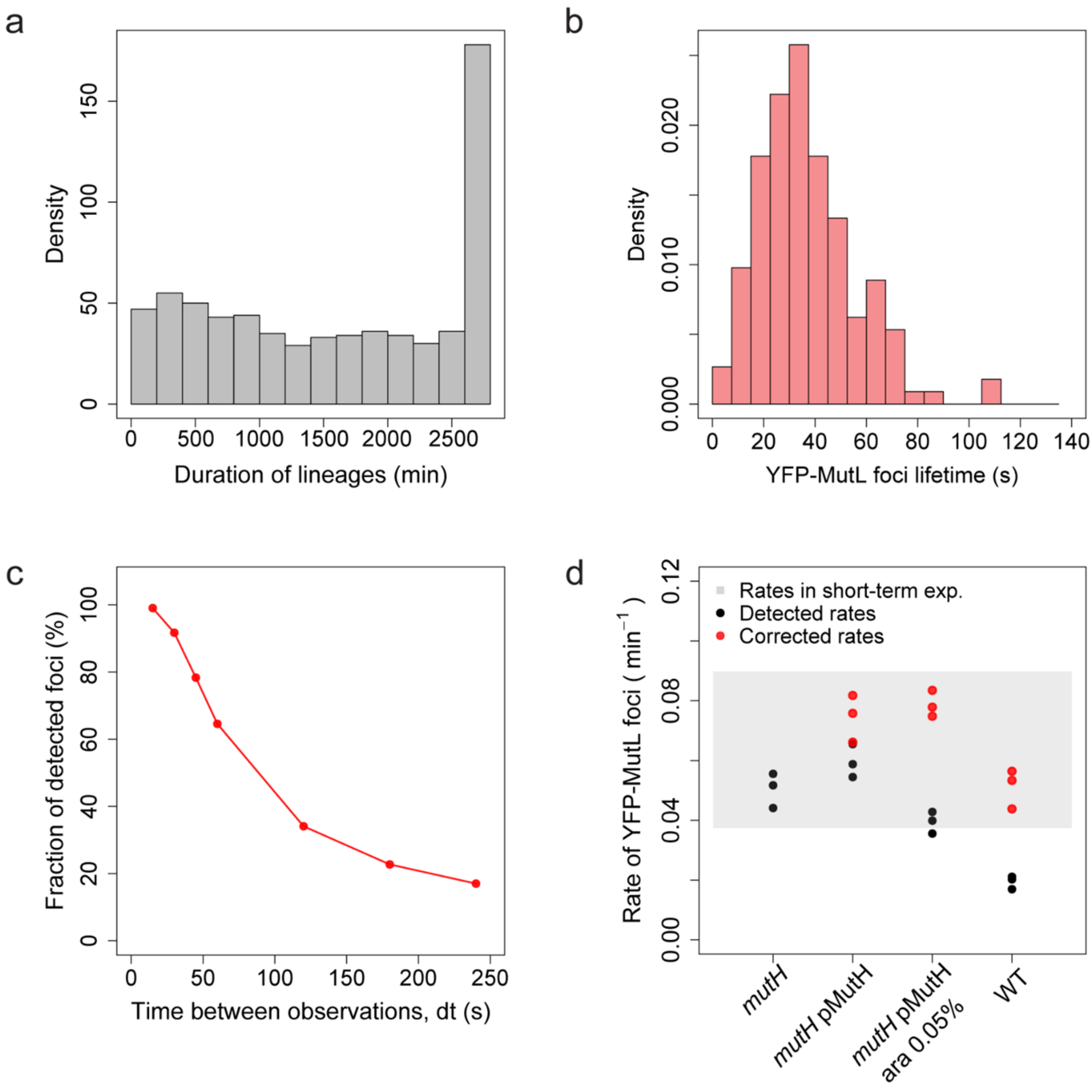
a
Density
0
50
100
150
0
500
1000
1500
2000
2500
Duration of lineages (min)
b
Density
0.000
0.010
0.020
0
20
40
60
80
100
120
140
YFP-MutL foci lifetime (s)
c
Fraction of detected foci (%)
0
20
40
60
80
100
0
50
100
150
200
250
Time between observations, dt (s)
d
Rates in short-term exp.
Detected rates
Corrected rates
Rate of YFP-MutL foci (min⁻¹)
0.00
0.04
0.08
0.12
mutH
mutH pMutH
mutH pMutH ara 0.05%
WT

Supplementary Figure 5. Threshold to distinguish between long-lived and short-lived YFP-MutL foci.

The result of the method used to determine the threshold between short-lived and long-lived YFP-MutL foci (see dedicated section in Supplementary Note 1). The lifetimes were measured in frames. Three *mutH* pMutH experiments (LB) were pooled together (n=1027). The magenta and blue curves represent the final best exponential and Gaussian fits, respectively, obtained as the output of the method. The vertical red line indicates the intersection point between the two curves, which sets the final threshold at 7 frames. Source data are provided as a Source Data file.

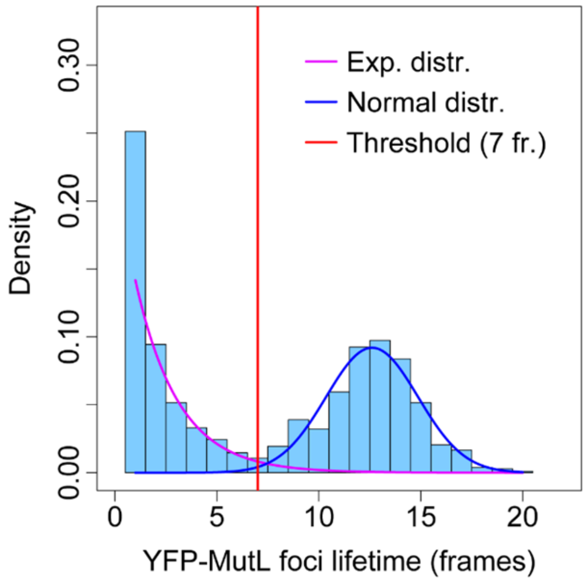

Supplementary Figure 6. A variation of +/- 2 minutes in the threshold on foci lifetimes doesn't change the conclusions on the mutation rates.

Equivalent of Figure 3b with three different thresholds used to determine the long-lived foci. Comparison of the mutation rate of strains WT, *mutH* pMutH with two different levels of induction (*mutH* pMutH ara 0.05% and *mutH* pMutH) and *mutH*, obtained using MA + WGS (dark grey) and long-lived YFP-MutL foci determined with the three different thresholds set at 6 frames (magenta), 7 frames (light grey, same as in Figure 3b), 8 frames (in cyan) (See Supplementary Note 1 section on the variation of the threshold of +/- 2 minutes). The bars represent the average of individual experiments (dots). The values of MA+WGS for the WT strain are from Lee et al., [4], for all the other conditions 3 MA+WGS experiments have been performed. For YFP-MutL foci experiments, n = 3 for WT, *mutH* pMutH and *mutH* ; n = 2 for *mutH* pMutH ara 0.05%; for MA+WGS experiments, n = 3 for *mutH* and *mutH* pMutH ara 0.05%, n = 4 for *mutH* pMutH and n = 2 for WT. Source data are provided as a Source Data file.

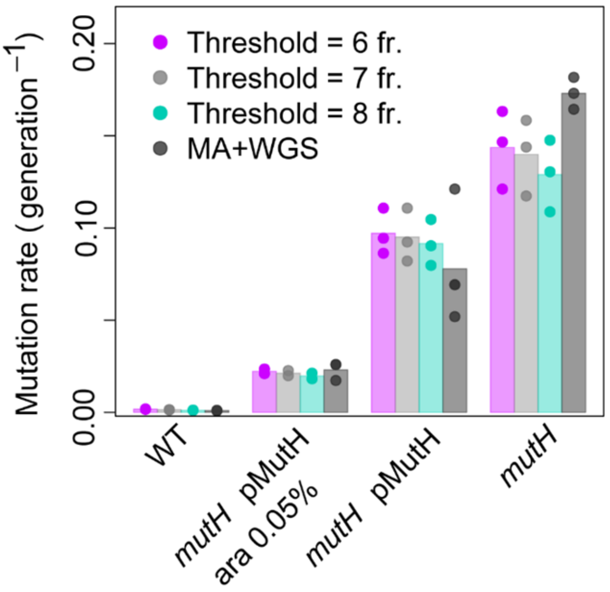

Supplementary Figure 7. Comparisons of distributions of cell length, growth rate, and foci lifetime in experiments where Dam and MutH levels were modulated.

The analysis was conducted on a subset of cells that were selected based on their length and growth rate according to a specific procedure described in the Supplementary Note 1 section on YFP-MutL foci in cells with varying levels of Dam and MutH. From left to right, cell length, cell growth rate and YFP-MutL foci lifetime for all the long-term experiments performed with **a)** WT PLtetO1-*dam* (n = 3), WT (n = 3), *mutH* (n = 3) and **b)** WT pMutH (n = 3), WT (n = 3) and *dam* (n = 4) cells. Source data are provided as a Source Data file.

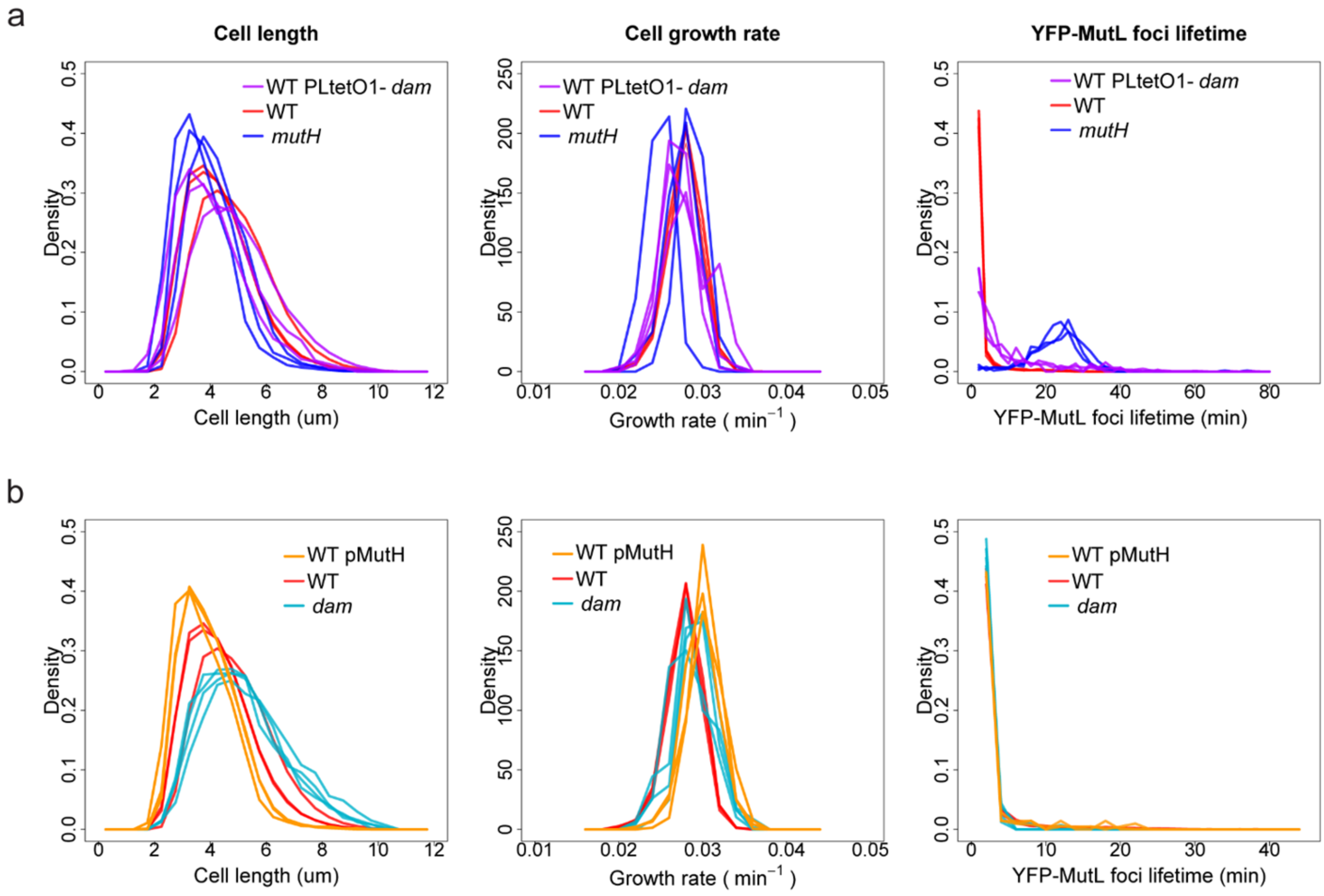

Supplementary Figure 8. Changing the threshold for selecting cells in the analysis of Dam and MutH competition does not alter the conclusions.

Equivalent of Figure 4a and 4b with the data selected with a more stringent criterion: cells (along with their mother and daughter) were discarded if either i) at any moment of their cell cycle they reach a length of at least ($\mu + 2\sigma$) = 8.5 µm, (μ and σ are the average and standard deviation of distribution of WT cell lengths) or ii) have growth rates outside the range $G = (\mu - 2\sigma, \mu + 2\sigma)$ (μ and σ are the average and standard deviation of distribution of WT growth rates, see Supplementary Note 1 section on YFP-MutL foci in cells with varying levels of Dam and MutH.). **a)** Rate of long-lived YFP-MutL foci for *mutH* (blue, n = 3), WT PLtetO1-*dam* (violet, n = 3), WT (red, n = 3), WT pMutH (yellow, n = 3) and *dam* (cyan, n = 4) (inset shows the zoom in for the last 3 conditions). **b)** Distribution of foci lifetimes of one representative experiment for each condition shown in a). Source data are provided as a Source Data file.

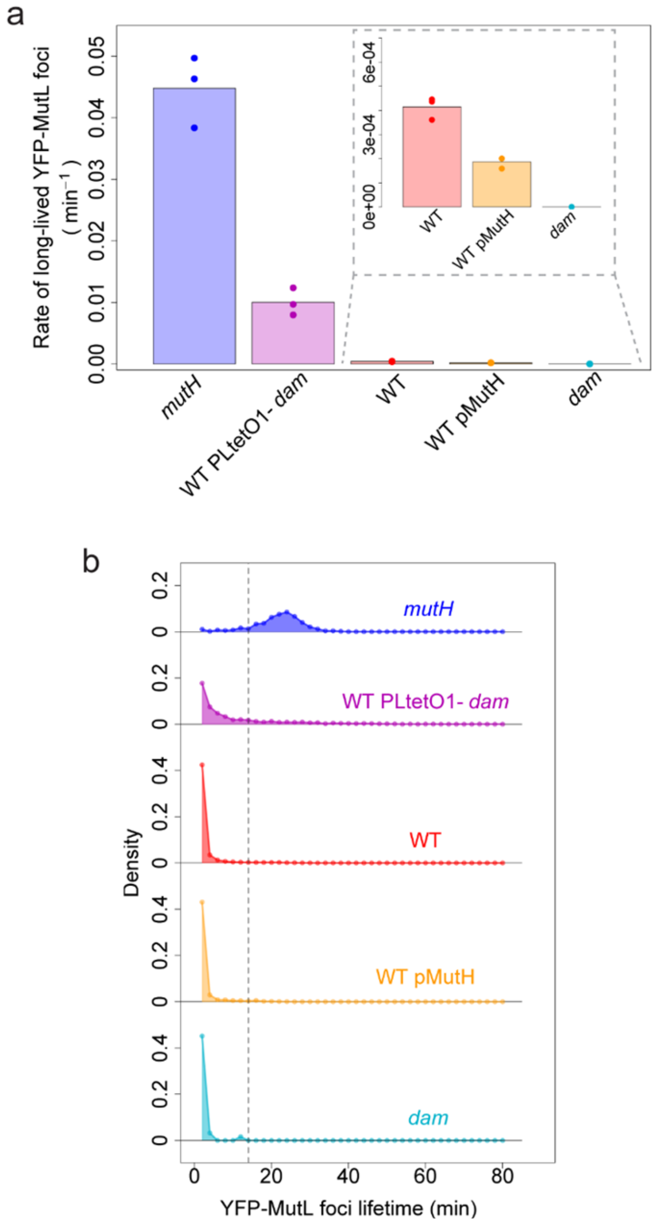
a
Rate of long-lived YFP-MutL foci
(min⁻¹)
0.00
0.01
0.02
0.03
0.04
0.05
0e+00
3e-04
6e-04
WT
WT pMutH
dam
mutH
WT PLtetO1- dam
WT
WT pMutH
dam
b
Density
mutH
WT PLtetO1- dam
WT
WT pMutH
dam
0
0.2
0.4
0
20
40
60
80
YFP-MutL foci lifetime (min)

Supplementary Figure 9. Cell growth rate as a function of replicative age.

The average cell growth rate was analysed as a function of replicative age, i.e., the number of cell divisions or generations, in the three WT experiments. Each experiment is represented by a distinct colour. All lineages were included in the analysis and their duration limited to 100 generations (see Supplementary Table 1 for total number of cells per experiment). The solid lines represent the average per-generation growth rate. The shaded area depicts 95% confidence interval. The black line represents the average growth rate. Source data are provided as a Source Data file.

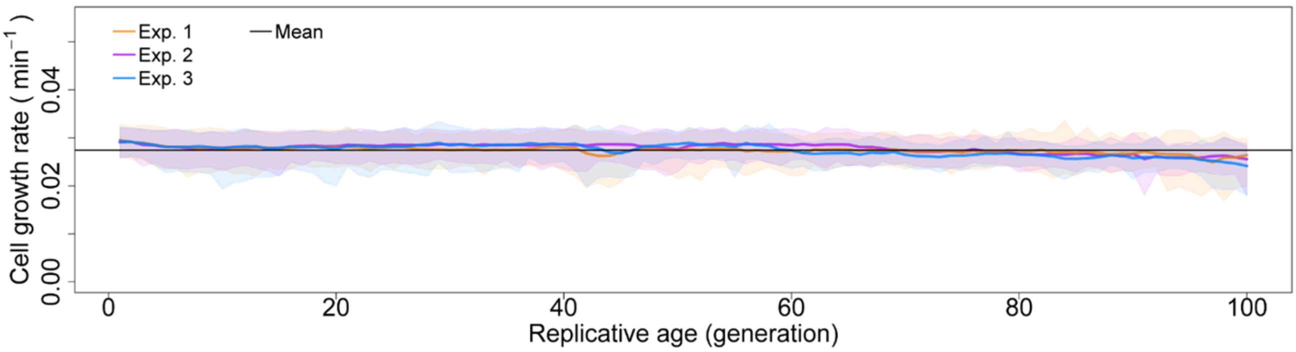

Supplementary Figure 10. Rates of YFP-MutL foci as a function of replicative age.

All lineages were included in the analysis and their duration limited to 100 generations. **a)** Rate of all YFP-MutL foci as a function of replicative age. Each colour corresponds to a single WT experiment. Dots are obtained by binning by 10 generations. The horizontal black line is the average of all the data. No significant difference between the first and the last 10 generations (two-sided Welch's t-test p = 0.91). **b)** Analogous to a), but focusing only on long-lived YFP-MutL foci. No significant difference between the first and the last 10 generations (two-sided Welch's t-test p = 0.74). Source data are provided as a Source Data file.

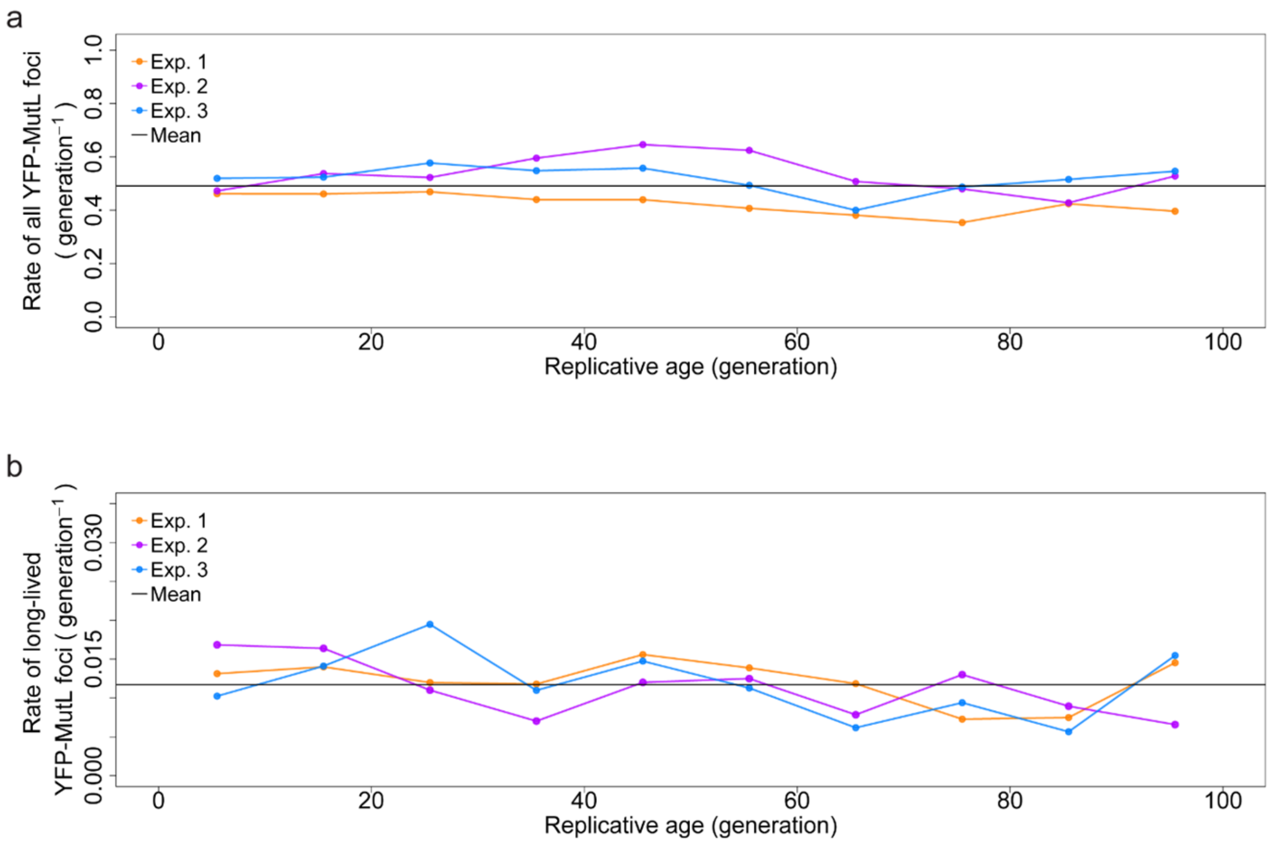

Supplementary Figure 11. Correction of the bias in the Cumulative Distribution Function (CDF) of inter-arrival times.

a). A schematic representation describes the procedure employed to correct the bias in the CDF of inter-arrival times and described in a dedicated section in Supplementary Note 1. The example shows 4 mother cell lineages (rectangular boxes called $M_{1,..4}$) observed over time from time 0 to times $W_{1,...4}$. Orange dots represent the occurrence of foci at specific times t_{ij} (i = 1 ,..4; j = 1,..n.). The red segments depict the foci inter-arrival times ($d_{i,j}$). For each discrete value of x, the size of the observation window W_i is reduced by x (grey shaded area) to calculate CDF(x). τ_i, δ_i, D_x and T_x are defined in the dedicated section in Supplementary Note 1. In the shown example, the value of the CDF(x) is $P(d \leq x) = D_x/T_x = 0.5$. **b)** The comparison is presented between the biased (blue), corrected (grey) and analytical (black) CDFs of inter-arrival times from 300 simulations of a Poisson process with a rate of $\Lambda = 0.00047$ min^{-1} (average rate of long-lived YFP-MutL foci in the three long-term WT experiments). Each simulation comprises 300 lineages, with each lineage observed for a time window W randomly drawn from the distribution of lineage durations in the three WT experiments (Supplementary Figure 4a). The solid blue and grey lines represent the average over the simulations, and the shaded areas represent 95% confidence interval of the simulations. The black line is the analytical expectation for a Poisson process with rate Λ and accounting for discrete observations. **c)** The comparison between the simulated CDF (in grey, same as in c)), the analytical expectation (in black, same as in b)) and the experimental data (orange, violet, light blue representing the three experiments, same as in Figure 6d). Source data are provided as a Source Data file.

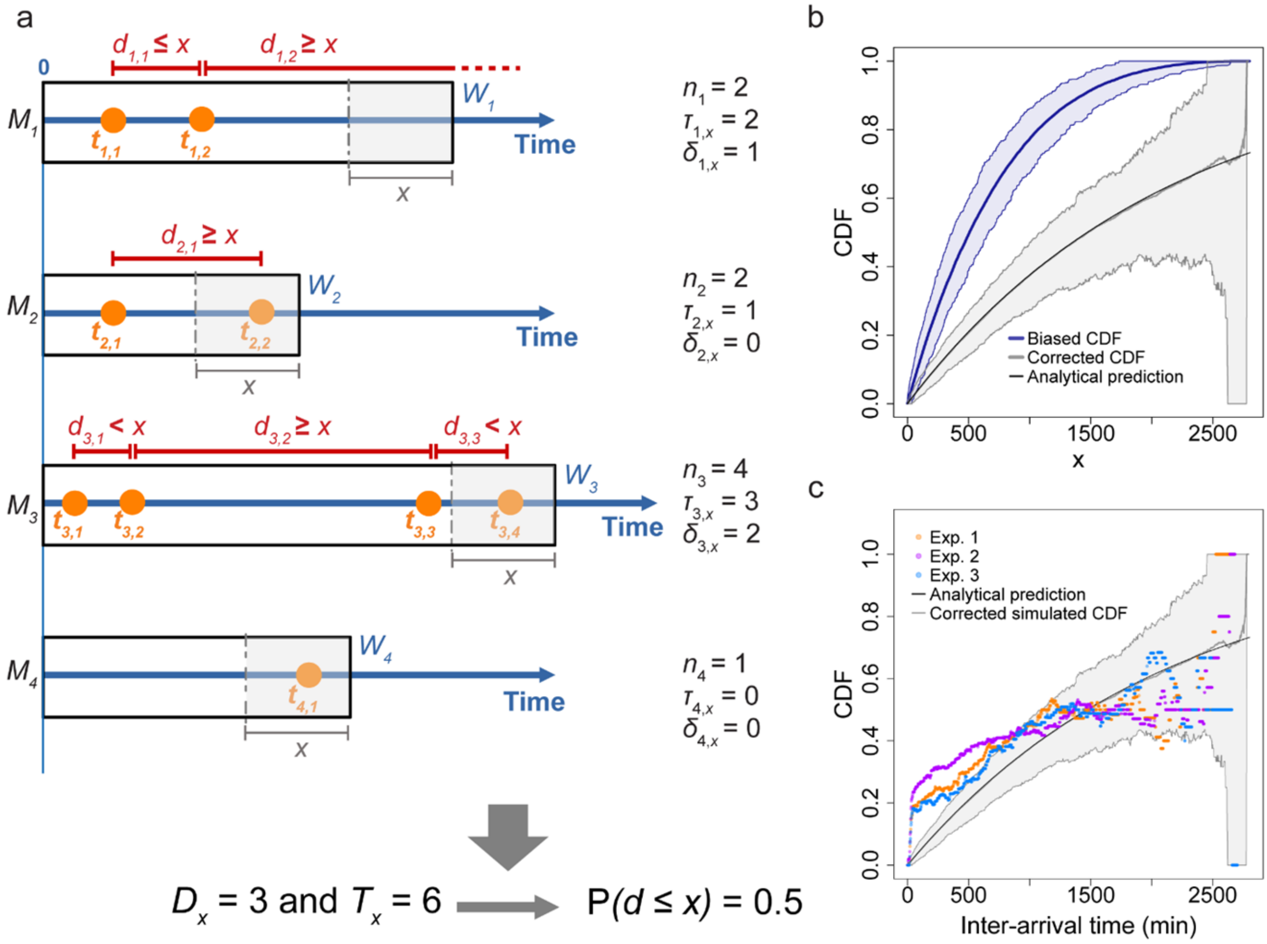
a
b
c
$d_{1,1} \le x$
$d_{1,2} \ge x$
$d_{2,1} \ge x$
$d_{3,1} < x$
$d_{3,2} \ge x$
$d_{3,3} < x$
Time
$n_1 = 2$
$\tau_{1,x} = 2$
$\delta_{1,x} = 1$
$n_2 = 2$
$\tau_{2,x} = 1$
$\delta_{2,x} = 0$
$n_3 = 4$
$\tau_{3,x} = 3$
$\delta_{3,x} = 2$
$n_4 = 1$
$\tau_{4,x} = 0$
$\delta_{4,x} = 0$
$D_x = 3$ and $T_x = 6$
$P(d \le x) = 0.5$
CDF
Biased CDF
Corrected CDF
Analytical prediction
x
Exp. 1
Exp. 2
Exp. 3
Analytical prediction
Corrected simulated CDF
Inter-arrival time (min)

Supplementary Figure 12. Confidence intervals of the Cumulative Distribution Function of the inter-arrival times for the three WT experiments.

The dots represent the experimental data (same as Figure 6d), shaded areas correspond to 95% confidence interval obtained through bootstrap method (see last section of Supplementary Note 1). Source data are provided as a Source Data file.

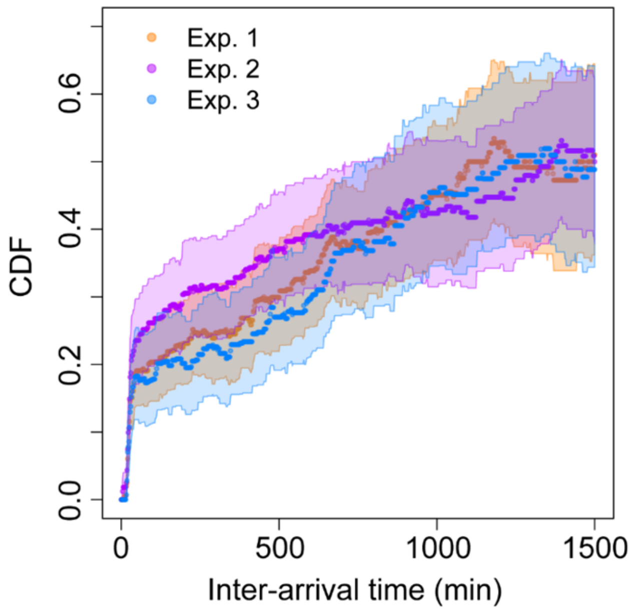

Supplementary Figure 13. A variation of +/- 2 minutes in the threshold value on foci lifetimes doesn't change the conclusions on the temporal dynamics of long-lived YFP-MutL foci.

Equivalent of Figure 6d with three different thresholds used to define the long-lived foci. Comparisons of the Cumulative Distribution Function (CDF) (empirical and analytical prediction) of long-lived YFP-MutL foci determined with different thresholds for one representative WT experiment. The chosen values for the thresholds are: 6 frames (magenta), 7 frames (orange as in Figure 6d) and 8 frames (cyan) (see dedicated section in Supplementary Note 1). Dots represent the empirical CDF corrected for the bias (see Supplementary Note 1 section on CDF correction for the bias) and lines represent the analytic prediction that accounts for discrete observations. Source data are provided as a Source Data file.

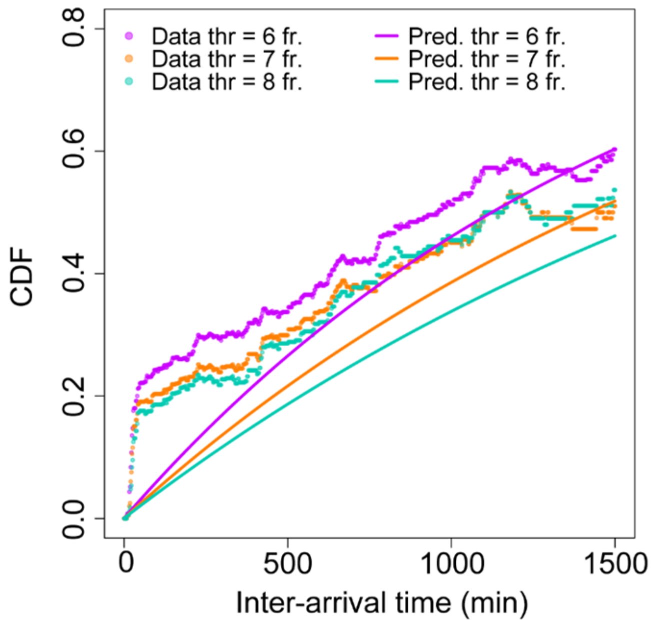

4. Supplementary tables

Supplementary Table 1. Number of cells and foci analysed in Figure 5b, Supplementary Figures 9 and 10.

Total number (N) of cells, all and long-lived foci analysed for each WT experiment.

**Experiment**	**N cells**	**N all YFP-MutL foci**	**N long-lived YFP - MutL foci**
WT1	15746	6834	197
WT2	13684	7370	163
WT3	11609	6053	141

Supplementary Table 2. Rate of YFP-MutL foci estimated from short-term experiments.

μ: mean rate of YFP-MutL foci appearing in frames 2-6; s_μ = 2* SEM (Standard Error of the Mean). All cells are analysed in each experiment. Source data are provided as a Source Data file.

Condition	$\mu \pm s_\mu$ (min^{-1})	N foci
WT 1	0.080 ± 0.014	132
WT 2	0.061 ± 0.015	64
WT 3	0.054 ± 0.013	70
mutH 1	0.064 ± 0.022	33
mutH 2	0.052 ± 0.014	57
MF1R 1	0.052 ± 0.009	134
MF1R 2	0.083 ± 0.013	158
MF1R ara 0.1% 1	0.195 ± 0.027	217
MF1R ara 0.1% 2	0.155 ± 0.015	427
MF1R ara 0.5% 1	0.60 ± 0.11	135
MF1R ara 0.5% 2	0.56 ± 0.08	205

Supplementary Table 3. Comparison of the rate of YFP-MutL foci for the data shown in Figure 1d.

The comparisons were performed using two-sided Welch's t-test.

Conditions tested	p-value
WT – *mutH*	0.53
WT – MF1R	0.91
MF1R – MF1R ara 0.1%	0.06
MF1R ara 0.1% – MF1R ara 0.5%	0.006

Supplementary Table 4. Number of YFP-MutL foci analysed in Figure 1e and Supplementary Figure 1.

Condition	N foci
WT 1	263
WT 2	121
WT 3	160
WT Pnat 1	76
WT Pnat 2	136

WT Pnat 3	46
mutH 1	272
mutH 2	101
MF1R 1	186
MF1R 2	83
MF1R ara 0.1% 1	161
MF1R ara 0.1% 2	350
MF1R ara 0.5% 1	127
MF1R ara 0.5% 2	212

Supplementary Table 5. Comparisons between the results obtained through MA+WGS for *mutS* and MF1R*mutS* at different levels of induction (Supplementary Figure 3).

The comparisons were performed using two-sided Welch's t-test.

Tested conditions	p-values
mutS – MF1R *mutS*	0.13
mutS – MF1R *mutS* ara 0.1%	$1.6*10^{-4}$
mutS – MF1R *mutS* ara 0.5%	$8*10^{-3}$

Supplementary Table 6. Results of simulations estimating the fraction of lost events due to discrete observations in long-term experiments.

Results of the simulations performed to correct the rates of YFP-MutL foci accounting for discrete observations. The 500 simulations have been performed as described in dedicated section of Supplementary Note 1 with dt = 2 minutes. *F* is the ratio between the mean short-lived foci lifetimes (< 16 minutes) of the analysed condition and that of WT. The data in the last column are the averages +/- 2*SEM of the results of 500 simulations.

Condition	Mean short-lived foci lifetime (minutes)	*F*	N data in the distribution (Fig. S5B)	Fraction of detected events (%)
WT	2.6	1	150	(34.07 ± 0.02)
mutH pMutH ara 0.05%	3.0	1.2	148	(39.87 ± 0.03)
mutH pMutH	4.2	1.6	140	(50.20 ± 0.04)

Supplementary Table 7. Fraction of long-lived YFP-MutL foci after correction for discrete observations.

Number of detected long-lived YFP-MutL foci, number of all foci before and after the correction for loss due to the 2 minutes discrete observations and fraction of long-lived foci over the total number of foci after the correction.

Condition	N detected long-lived foci	N all detected foci	N corrected foci	% long lived foci (after correction)
WT1	203	7060	18297	1.1
WT2	165	7632	20452	0.8
WT3	145	6241	16500	0.9
mutH pMutH ara 0.05% 1	1039	188	2024	9
mutH pMutH ara 0.05% 2	1190	178	2317	8

mutH pMutH 1	137	280	362	38
mutH pMutH 2	216	406	508	43
mutH pMutH 3	181	341	415	44

Supplementary Table 8. Comparison of mutation rates estimates obtained through MA+WGS and YFP-MutL foci for the data shown in Figure 3b.

The comparisons were performed using two-sided Welch's t-test.

Condition	p-value
WT	0.18
mutH pMutH ara 0.05%	0.62
mutH pMutH	0.37
mutH	0.09

Supplementary Table 9. Rates of YFP-MutL foci in long-term experiments.

The table shows the rates of all (μ_{all}) and long-lived (μ_{long}) YFP-MutL foci, along with their error (2*SEM) and sample sizes (number of cells, total detected foci and long-lived foci) for the data presented in Supplementary Figure 7, Figure 4a and b. Source data are provided as a Source Data file.

Condition	μ_{all} (min^{-1})	μ_{long} (min^{-1})	N cells	N all foci	N long-lived foci
mutH 1	(0.043 ± 0.003)	(0.038 ± 0.003)	676	713	626
mutH 2	(0.052 ± 0.006)	(0.047 ± 0.006)	219	314	286
mutH 3	(0.056 ± 0.006)	(0.050 ± 0.006)	198	288	258
WT 1	(0.0160 ± 0.0004)	(4.6 ± 0.7) $*10^{-4}$	14629	5874	168
WT 2	(0.0195 ± 0.0005)	(3.8 ± 0.7) $*10^{-4}$	13046	6362	125
WT 3	(0.0192 ± 0.0005)	(4.5 ± 0.8) $*10^{-4}$	11019	5375	127
WT pMutH 1	(0.0099 ± 0.0005)	(2.3 ± 0.8) $*10^{-4}$	5854	1399	32
WT pMutH 2	(0.0048 ± 0.0016)	(1.37 ± 2.7) $*10^{-4}$	291	35	1
WT pMutH 3	(0.0044 ± 0.0009)	(1.7 ± 1.7) $*10^{-4}$	949	103	4
WT PLtetO1-dam 1	(0.057 ± 0.003)	(0.0136 ± 0.0015)	917	1409	337
WT PLtetO1-dam 2	(0.043 ± 0.007)	(0.010 ± 0.003)	138	164	38
WT PLtetO1-dam 3	(0.058 ± 0.013)	(0.009 ± 0.005)	61	75	12
dam 1	(0.013 ± 0.003)	0	203	68	0
dam 2	(0.023 ± 0.006)	0	87	52	0
dam 3	(0.019 ± 0.006)	0	85	40	0
dam 4	(0.015 ± 0.005)	0	96	34	0

Supplementary Table 10. Contingency tables for Barnard's unconditional test performed on data of Figure 5b.

	First 10 generations	Last 10 generation
N long-lived foci	88	25
N short-lived foci	3077	995

Supplementary Table 11: Bacterial strains and plasmids

Strain/plasmid	Genotype	Source
MG1655 6300		GSC
MG1655		Matic lab
63ME120R	MG1655 6300, but *lacZ::yfp-mutL*-frt, *mutL*::frt, *att*Tn*7*::pRNA1-*tdCherry*	[1]
63ME121R	63ME120R, but *mutH*::*cam*	[1]
MF1	MG1655 6300, but *att*P21::*araC*-PBad-*dnaQ926*	[1]
MF1R	MF1, but *lacZ::yfp-mutL*-frt, *mutL*::frt, *att*Tn*7*::pRNA1-*tdCherry*	This study
MVEC249	MG1655 6300, but *mutS::spec/strep*	This study
MVEC253	MF1R, but *mutS::spec/strep*	This study
63ME130R	63ME120R, but pBAD24MutH	This study
63ME132R	63ME121R, but pBAD24MutH	This study
MVEC205	63ME120R, but *dam*::cat	This study
MVEC197	63ME120R, but *att*P21::PLtetO1-*dam*	This study
ME131	MG1655, but *mutL::yfp-mutL-cam*	[8]
63ME134R	MG1655 6300, but *mutL::yfp-mutL-cam*, *att*Tn*7*::pRNA1-*tdCherry*	This study
pBAD24MutH		[6]
pNDL32		Paulsson lab

Supplementary References

1. Robert, L. *et al.* Mutation dynamics and fitness effects followed in single cells. *Science* **359**, 1283–1286 (2018).
2. Wang, P. *et al.* Robust Growth of Escherichia coli. *Curr. Biol.* **20**, 1099–1103 (2010).
3. Deatherage, D. E. & Barrick, J. E. Identification of mutations in laboratory evolved microbes from next-generation sequencing data using breseq. *Methods Mol. Biol. Clifton NJ* **1151**, 165–188 (2014).
4. Lee, H., Popodi, E., Tang, H. & Foster, P. L. Rate and molecular spectrum of spontaneous mutations in the bacterium Escherichia coli as determined by whole-genome sequencing. *Proc. Natl. Acad. Sci.* **109**, E2774–E2783 (2012).
5. St-Pierre, F. *et al.* One-Step Cloning and Chromosomal Integration of DNA. *ACS Synth. Biol.* **2**, 537–541 (2013).
6. Elez, M., Radman, M. & Matic, I. Stoichiometry of MutS and MutL at unrepaired mismatches in vivo suggests a mechanism of repair. *Nucleic Acids Res.* **40**, 3929–3938 (2012).
7. Datsenko, K. A. & Wanner, B. L. One-step inactivation of chromosomal genes in Escherichia coli K-12 using PCR products. *Proc. Natl. Acad. Sci.* **97**, 6640–6645 (2000).
8. Elez, M., Robert, L. & Matic, I. Method for Detecting and Studying Genome-Wide Mutations in Single Living Cells in Real Time. *Methods Mol. Biol. Clifton NJ* **1736**, 29–39 (2018).
